# Formulation-dependent dissolution and bioaccessibility of curcuminoids and (S)-ar-turmerone from eight commercial turmeric extract- and curcumin-containing dietary supplements

**DOI:** 10.1080/13880209.2026.2631826

**Published:** 2026-02-23

**Authors:** Bill J. Gurley, Catherine M. Gurley, Kumar Katrugunta, Bharathi Avula, Ikhlas A. Khan, Amar Chittiboyina, Philip W. Melchert, John S. Markowitz

**Affiliations:** ^a^ National Center for Natural Products Research, School of Pharmacy, University of Mississippi, University, MS, USA; ^b^ Department of Pharmacotherapy and Translational Research, University of Florida, Gainesville, FL, USA

**Keywords:** Curcumin, turmeric dietary supplements, biorelevant dissolution, bioaccessibility, dosage-form performance

## Abstract

**Context:**

Curcumin-containing dietary supplements are widely marketed with claims of enhanced bioavailability, despite well-recognized limitations related to poor aqueous solubility, chemical instability, and extensive first-pass metabolism. Comparisons among commercially available products using physiologically relevant performance metrics remain limited.

**Objective:**

To systematically evaluate disintegration, dissolution, and bioaccessibility of curcuminoids and (S)-ar-turmerone from a cross section of commercially available turmeric dietary supplements under fasted- and fed-state biorelevant conditions.

**Materials and Methods:**

Eight marketed turmeric supplements representing diverse formulation strategies were assessed for disintegration and dissolution using USP-aligned methods in fasted- and fed-state simulated gastric and intestinal media (FaSSGF, FaSSIF, FeSSGF, FeSSIF). Bioaccessible concentrations, quantities, and dose fractions of curcuminoids and (S)-ar-turmerone were quantified after 3 h.

**Results:**

All products exhibited poor dissolution overall, with no formulation achieving greater than 40% total release. Dissolution was lowest under fasted-state conditions and improved modestly in fed-state gastric media, reflecting the influence of lipid content. Products with higher curcuminoid loads per capsule generated greater absolute bioaccessible concentrations despite poor release efficiency, whereas a phytosome formulation achieved superior release despite a lower dose. Several products marketed as “enhanced” formulations demonstrated poor disintegration and low bioaccessibility.

**Discussion:**

These findings indicate that bioaccessible concentration is governed jointly by dosage-form performance and curcuminoid dose loading, and that plasma exposure metrics dominated by conjugated metabolites may not reliably reflect formulation performance.

**Conclusion:**

Commercial turmeric supplements exhibit substantial limitations in biorelevant disintegration and dissolution. Superior *in vitro* release is a prerequisite – but not a guarantee – for enhanced systemic exposure, underscoring the need for cautious interpretation of bioavailability claims.

## Introduction

Turmeric (*Curcuma longa*), a rhizomatous perennial in the ginger family (*Zingiberaceae*), has held a prominent role in South Asian and Middle Eastern culinary and medicinal traditions for centuries. Long revered in Ayurveda and traditional Chinese medicine for its anti-inflammatory, digestive, and wound-healing properties, turmeric has in recent decades undergone a resurgence in global interest as a functional food and natural health product (Engels 2009). This modern revival is largely driven by the bioactive compounds found in turmeric rhizomes – chiefly the curcuminoids – and has been accompanied by a proliferation of turmeric-containing dietary supplements across global markets. In the United States, turmeric has ranked among the top-selling herbal dietary supplements for at least five consecutive years, particularly within the mass-market retail sector, a trend that reflects both sustained consumer demand and continued scientific interest in its potential health benefits.

The major pharmacologically active constituents of turmeric include curcumin (diferuloylmethane) and its two naturally co-occurring analogs, demethoxycurcumin and bisdemethoxycurcumin. These three polyphenols, collectively known as curcuminoids, are responsible for turmeric’s characteristic yellow color and are widely credited with its antioxidant, anti-inflammatory, and potential chemopreventive effects (Kocaadam and Şanlier 2017). In addition to these nonvolatile polyphenols, turmeric also contains a group of aromatic sesquiterpenes known as turmerones, which are concentrated in the essential oil fraction and may possess complementary biological activities (Orellana-Paucar and Machado-Orellana 2022). However, the clinical translation of turmeric’s traditional uses into evidence-based outcomes has been hindered by the pharmacokinetic limitations of its primary constituents.

Curcumin and its congeners are highly lipophilic and exhibit poor aqueous solubility, chemical instability under physiological conditions, and extensive presystemic metabolism (Kotha and Luthria 2019). Structurally, curcumin features two ortho-methoxyphenol rings joined by a seven-carbon conjugated chain, a configuration that contributes to its low solubility in water and susceptibility to degradation at neutral or basic pH (Kotha and Luthria 2019). Following oral administration, curcuminoids are rapidly metabolized in the intestinal mucosa and liver through glucuronidation and sulfation, yielding conjugated metabolites that dominate systemic exposure, despite uncertainty regarding their intrinsic biological activity. As noted by Nelson and colleagues, unconjugated (free) curcumin is typically undetectable in plasma unless samples are enzymatically treated with β-glucuronidase (Nelson et al. 2017a). These combined barriers – limited solubility, low chemical stability, and rapid first-pass metabolism – severely restrict oral bioavailability and complicate therapeutic applications.

In response to the well-recognized biopharmaceutical limitations of curcumin, a growing number of turmeric supplement manufacturers have adopted novel formulation strategies intended to enhance absorption (Tabanelli et al. 2021). These include lipid-based emulsions, solid lipid particles, nanoparticles, micelles, liposomes, phospholipid complexes (e.g., phytosomes), and absorption enhancers such as piperine. While such approaches may improve systemic exposure under select conditions, they are frequently supported by limited in-house data and rarely evaluated in direct, head-to-head comparisons across multiple marketed products (Jamwal 2018). As a result, claims of “enhanced absorption” or “bioavailability-boosted” delivery platforms are often advanced without corresponding evidence of dosage-form performance, a foundational determinant of bioaccessibility.

For compounds such as curcumin, which exhibit both low aqueous solubility and low permeability, dosage-form disintegration and dissolution are commonly rate-limiting steps in systemic absorption. Despite this, relatively little attention has been paid to the performance of commercially available turmeric supplements under physiologically relevant conditions. Most prior dissolution studies have focused on isolated curcumin preparations or experimental matrices tested in simplified buffer systems that do not recapitulate the complex environment of the human gastrointestinal tract. In contrast, biorelevant dissolution media – including fasted- and fed-state simulated gastric and intestinal fluids (FaSSGF, FeSSGF, FaSSIF, and FeSSIF) –incorporate bile salts, phospholipids, and pH conditions that more closely mimic *in vivo* fasted and postprandial states (Kleberg et al. 2010; Klein 2010; Dressman 2014).

Given the expanding use of novel delivery technologies in turmeric supplements and the prominent marketing claims accompanying them, there is a clear need to evaluate how real-world products perform with respect to disintegration and dissolution in biorelevant media. The present study addresses this gap by systematically assessing eight commercially available turmeric-containing dietary supplements encompassing a broad range of delivery technologies, including standard extract tablets, phytosome complexes (4297PR), formulations incorporating bioavailability enhancers such as piperine, and engineered carrier systems such as hydrophilic polymer-based dispersions (4819PR), protein–polyphenol conjugates (4664PR and 4663PR), and a curcuminoid formulation co-delivered with an ar-turmerone–rich essential oil fraction (4818PR) (Gurley 2011; Kurita and Makino 2013; Liu et al. 2016; Ma et al. 2019; Zheng and McClements 2020). By evaluating both disintegration and curcuminoid dissolution across multiple fasted- and fed-state biorelevant media, this work enables direct comparisons across formulations and provides foundational data for interpreting pharmacokinetic relevance, clinical potential, and regulatory considerations.

## Materials and methods

### Supplements

Eight turmeric extract- or purified curcumin-containing supplements were purchased from local retailers or from internet sources. Product formulations ranged from a tableted standard turmeric extract to a selection of encapsulated novel delivery systems designed to enhance curcuminoid bioavailability. To avoid brand-specific bias and to focus on comparative dosage-form performance, all commercially available turmeric dietary supplements evaluated in this study were assigned anonymized product codes. Product identities are disclosed only by formulation characteristics and label-declared composition. A description of the coded products, their delivery platform, and biohancement strategy is provided in Table 1.

**Table 1.** Commercially available turmeric extract- and curcumin-containing dietary supplements evaluated for disintegration and dissolution performance.

Product Code	Delivery Platform	Bioenhancement Strategy
4295PR	Tablet (turmeric extract)	None (baseline control)
4296PR	Capsule (turmeric extract)	Addition of black pepper extract (piperine) to enhance absorption
4297PR	Capsule (curcumin phytosome)	Phospholipid complexation (Meriva^®^ technology using lecithin)
4299PR	Dual chamber capsule (turmeric extract + turmeric essential oil)	Combination of turmeric extract and turmeric essential oil (AR-turmerone-rich fraction)
4663PR	Capsule (protein–polyphenol conjugate)	N-acetylcysteine–curcumin complex (NAC-Curcumin Complex^™^ + Plantisorb Curcumin^™^)
4664PR	Capsule (protein–polyphenol conjugate)	Whey protein–curcumin complex (UltraCur LPS^™^ platform)
4818PR	Capsule (curcuminoid + turmeric essential oil)	Bioenhancement *via* AR-turmerone-rich essential oil co-formulation
4819PR	Capsule (hydrophilic carrier dispersion)	Proprietary water-dispersible carrier system (includes excipients + curcuminoid extract)

### Biorelevant media

The biorelevant dissolution media used in this study – fasted state simulated gastric fluid (FaSSGF), fed state simulated gastric fluid (FeSSGF), fasted state simulated intestinal fluid (FaSSIF), and fed state simulated intestinal fluid (FeSSIF) – were prepared according to the manufacturer’s instructions (Biorelevant.com, Biorelevant, London, UK). While the exact component concentrations of FeSSGF remain proprietary, its formulation is known to include 62.5 g/L of fats and bile salts. The resulting pH values for the media were 1.7 (FaSSGF), 6.5 (FaSSIF), 5.0 (FeSSIF), and 4.5 (FeSSGF). FaSSGF and FaSSIF simulate the fasted-state gastric and intestinal environments, respectively, whereas FeSSGF and FeSSIF represent postprandial (fed-state) conditions. The quantities of natural surfactants and lipid components used in each medium, as disclosed by the manufacturer, are summarized in Table 2.

**Table 2.** Biorelevant media used for curcumin stability, disintegration, and dissolution studies.

Medium	pH	Total Surfactant of fat content (g/L)	Composition Description
FaSSGF	1.7	0.06 g natural surfactants	Simulated fasted gastric fluid
FaSSIF	6.5	2.02 g natural surfactants	Simulated fasted intestinal fluid
FeSSIF	5.0	10.6 g natural surfactants	Simulated fed intestinal fluid
FeSSGF	4.5	62.5 g fats and bile salts	Simulated fed gastric fluid (undisclosed composition)

### Analytical methodology

The chromatographic method employed for quantifying curcuminoids and (S)-ar-turmerone was adapted from the validated ultra-high performance liquid chromatography (UHPLC) procedure reported previously (Avula et al. 2012). Analyses were performed using a Waters Acquity UPLC system (Waters Corporation, Milford, MA, USA) equipped with a photodiode array (PDA) detector. Chromatographic separation was achieved on an Acquity BEH Shield RP18 column (2.1 × 100 mm, 1.7 μm particle size), with the column temperature maintained at 40 °C throughout the analysis. The mobile phase consisted of water (solvent A) and acetonitrile (solvent B), each containing 0.05% formic acid to aid ionization and peak shape. The gradient elution program was modified from the original method to optimize peak resolution and accommodate the matrix complexity of biorelevant media, with a flow rate of 0.25 mL/min.

To prevent sample carryover, a strong needle wash (acetonitrile/water, 95:5 v/v) and a weak seal wash (acetonitrile/water, 10:90 v/v) were used between injections. Samples were filtered through 0.22 μm PTFE syringe filters and degassed prior to injection; the injection volume was 2 μL. Quantitative detection of curcuminoids – curcumin, demethoxycurcumin, and bisdemethoxycurcumin – was carried out at 420 nm, while (S)-ar-turmerone, when included in the sample matrix, was monitored at 240 nm. Data acquisition and processing were conducted using Empower 3.8 software (Waters Corporation, Milford, MA, USA).

The method offered excellent sensitivity, resolution, and reproducibility, and was capable of resolving all curcuminoid components even in complex matrices such as FaSSGF, FeSSGF, FaSSIF, and FeSSIF. Method performance characteristics, such as linearity, accuracy, precision, and detection limits for the target analytes, were previously validated according to ICH guidelines and demonstrated suitability for dietary supplement and botanical matrix analysis (Avula et al. 2012).

Stability studies were performed by a high-performance liquid chromatography system (Shimadzu Scientific Instruments, Columbia, MD, USA) coupled to an AB Sciex API 3000 Triple-Quadrupole Mass Spectrometer (Applied Biosystems, Foster City, CA, USA) equipped with a Heated-Source-Induced-Disassociation Device (HSID), (Ionics, Concord, ON, Canada) and equipped with Analyst 1.6.3 software (Sciex, Marlborough, MA, USA). In brief, chromatographic separation was achieved using a C18 reverse-phase analytic column (Aqua, 50 × 2.0 mm, 5 µm; Phenomenex Inc., Torrance, CA) utilizing an isocratic method for 6 min at a mobile phase of 65% organic (methanol) and 35% aqueous (0.1% formic acid in water) at a flow rate 0.25 mL/min. Further details regarding the assay and quantitation of curcumin (analyte) and phenacetin (internal standard) is further described in Melchert et al. (2025).

### Dosage form content analysis

Seven dosage forms from each product were analyzed for curcuminoid content – including curcumin, desmethoxycurcumin, and bisdesmethoxycurcumin – as well as (S)-ar-turmerone, using the UHPLC method described above. Each capsule or tablet was accurately weighed (∼50 mg), exhaustively extracted with 10 mL of methanol, and filtered prior to analysis. Detection was performed at 420 nm for curcuminoids and 240 nm for (S)-ar-turmerone. Quantification was based on external calibration curves generated from authentic reference standards, with final concentrations adjusted for average sample weight and dilution factor.

In addition to the curcuminoids, (S)-ar-turmerone was measured due to its potential role as an inhibitor of the ABCB1 efflux pump (P-glycoprotein) (Yue et al. 2012). Aromatic turmerones may enhance the intestinal absorption of curcumin by reducing its P-gp-mediated efflux, suggesting that some turmeric extracts may already possess intrinsic bioenhancing properties. Reported values for curcuminoid and turmerone content were compared with each product’s label to assess claim accuracy.

### Curcumin stability assessment in biorelevant media

To evaluate curcumin degradation under physiologically and experimentally relevant conditions, we examined the stability of both pure curcumin and curcumin derived from a standardized *Curcuma longa* extract in five aqueous media: FaSSGF), FaSSIF, FeSSIF, 50:50 methanol/water with 0.1% formic acid (v/v), and phosphate buffer (0.1 M, pH 7.4). The three biorelevant media were prepared according to protocols provided by Biorelevant.com (Biorelevant, London, UK), which yield the following final pH values: FaSSGF (pH 1.7), FaSSIF (pH 6.5), and FeSSIF (pH 5.0). All media were prepared fresh on the day of use and equilibrated to 37 °C before usage.

Per the method of Wang et al. curcumin stability was assessed using two source materials: pure curcumin (Sigma Aldrich) and a vouchered turmeric extract standardized to 95% curcuminoids (University of Mississippi Botanical Repository) (Wang et al. 1997). Each test medium was dosed with curcumin at a final concentration of approximately 100 µM of pure curcumin or 50 µM of curcumin in the ethanolic turmeric extract. For each condition, duplicate microcentrifuge tubes were maintained at 37 °C in a water bath. Samples were withdrawn at 0, 15, 30, 60, 90, and 120 min. Immediately upon removal, each aliquot was quenched with an equal volume of ice-cold acetonitrile containing 100 nM phenacetin (internal standard) to halt further degradation. Samples were then centrifuged at 16,100 g for 10 min at 4 °C, and the resulting supernatants were analyzed s *via* LC-MS/MS analysis as described above (Melchert et al. 2025).

### Disintegration testing

Disintegration testing was conducted in accordance with USP General Chapter <2040> Disintegration and Dissolution of Dietary Supplements, with modifications to accommodate testing in FaSSGF, FeSSGF, FaSSIF, and FeSSIF. (USP-NF <2040>; USP-NF, Curcuminoids Capsules; USP-NF, Curcuminoids Tablets). All media were prepared following the manufacturer’s instructions (Biorelevant, London, UK), with pH values of 1.7 (FaSSGF), 4.5 (FeSSGF), 6.5 (FaSSIF), and 5.0 (FeSSIF).

Testing was performed using an Agilent 100 disintegration apparatus (Agilent, Santa Clara, CA, USA) maintained at 37 °C. Each test used six dosage forms placed in the six-cylinder basket assembly. Fluted disks were employed to prevent capsule flotation, as permitted under <2040>. The apparatus operated at a basket cycle rate of 30 strokes per minute.

Complete disintegration was defined as the absence of intact capsule shells or undispersed turmeric extract within the basket. A 30-minute disintegration time was used as the standard pass criterion. Products that failed to disintegrate within 30 min were monitored for up to 90 min. Due to the opacity of FeSSGF, baskets were removed and visually inspected at 15-minute intervals.

### Dissolution testing

Dissolution studies were performed in accordance with USP general chapter <2040> Disintegration and Dissolution of Dietary Supplements and the Curcuminoids Capsules monograph, with modifications to allow testing in biorelevant media (USP-NF <2040>; USP-NF, Curcuminoids Capsules; USP-NF, Curcuminoids Tablets). Six replicates of each product were evaluated using USP Apparatus 2 (paddle method) at 100 rpm and 37 °C in an Agilent 708-DS dissolution system (Agilent Technologies, Santa Clara, CA, USA). Each vessel contained 900 mL of FaSSGF, FeSSGF, FaSSIF, or FeSSIF, prepared according to the manufacturer’s instructions as previously described.

For dosage forms that floated, spiral wire sinkers were used to ensure full immersion. Aliquots (1.0 mL) were withdrawn from each vessel at baseline (0 min) and at 15, 30, 45, 60, 90, 120, and 180 min and passed through a pre-saturated 0.45 µM GMF filter (Biorelevant, London, UK). Following each withdrawal, an equal volume (1.0 mL) of fresh, pre-warmed medium was added to maintain constant volume. The supernatants were then analyzed for curcuminoids and (S)-ar-turmerone content using the UHPLC method described above.

Percent release (or bioaccessible dose fraction) at 3 h was calculated as the quotient of the total measured bioaccessible curcuminoids plus (S)-ar-turmerone present in the dissolution medium, divided by the total curcuminoid plus (S)-ar-turmerone content per capsule, multiplied by 100. Bioaccessible concentrations and quantities therefore represent net recoverable phytochemical levels in solution at each time point and inherently reflect the combined effects of dosage-form disintegration, dissolution, and chemical degradation within each biorelevant medium. No correction was applied to account for degradation products or theoretical maximum release, as the intent of these metrics was to capture the fraction of material remaining potentially available for absorption under physiologically relevant conditions.

## Results

### Curcuminoid and (S)-ar-turmerone content relative to label claims

Tables 3 and 4 summarize the mean (± RSD) content of curcuminoids and (S)-ar-turmerone, respectively, for each of the eight turmeric-containing products analyzed. For curcuminoids, three products contained quantities below their stated label claims: 4818PR (−6%), 4299PR (−13%), and 4296PR (−21%). Two products exceeded their stated label amounts: 4819PR (+32%) and 4295PR (+3%). The remaining three products did not specify curcuminoid content on the label, precluding comparison.

**Table 3.** Curcuminoid content per capsule (mean ± SD) versus product label claim.

Product	Curcumin (mg/capsule)	Desmethoxycurcumin (mg/capsule)	Bisdesmethoxycurcumin (mg/capsule)	Total curcuminoids (mg/capsule)	Label claim (Total curcuminoids/capsule)
**4295PR**	404.5 ± 4.5	76.6 ± 4.1	9.2 ± 4.3	490.3 ± 4.3	475 mg
**4296PR**	305.8 ± 3.5	60.7 ± 4.0	9.0 ± 2.8	375.5 ± 3.4	475 mg
**4297PR**	70.9 ± 4.7	14.5 ± 3.8	1.8 ± 3.5	87.2 ± 4.0	No claim
**4299PR**	107.0 ± 2.9	21.1 ± 2.9	2.5 ± 1.8	130.6 ± 2.5	150 mg
**4663PR**	389.5 ± 2.6	6.1 ± 3.4	0.5 ± 4.8	396.1 ± 3.6	No claim
**4664PR**	101.5 ± 1.7	2.0 ± 2.4	0.2 ± 0.5	103.7 ± 1.5	No claim
**4818PR**	347.3 ± 1.5	74.0 ± 3.3	7.8 ± 2.9	444.8 ± 2.6	475 mg
**4819PR**	105.2 ± 1.1	23.6 ± 0.8	3.3 ± 3.2	132.1 ± 1.7	100 mg

**Table 4.** (S)-ar-Turmerone content per capsule (mean ± SD) versus product label claim.

Product	(S)-ar-turmerone (mg/capsule)	Label claim ((S)-ar-turmerone/capsule)
4295PR	0.5 ± 2.7	No claim
4296PR	2.0 ± 1.3	No claim
4297PR	20.2 ± 0.7	No claim
4299PR	15.9 ± 3.9	No claim
4663PR	0.1 ± 4.3	No claim
4664PR	0.1 ± 0.8	No claim
4818PR	15.7 ± 4.2	No claim
4819PR	ND	No claim

ND = not detected.

None of the tested products provided a label claim for (S)-ar-turmerone, although measurable quantities were detected in all but one formulation. Among products with detectable levels, (S)-ar-turmerone content varied markedly, ranging from 20.2 mg/capsule in 4297PR’s formulation to non-detectable levels in 4819PR. These findings suggest considerable variability in the inclusion or retention of volatile sesquiterpenes across turmeric extract preparations.

### Stability of curcumin in biorelevant and control media

**Figure 1.**
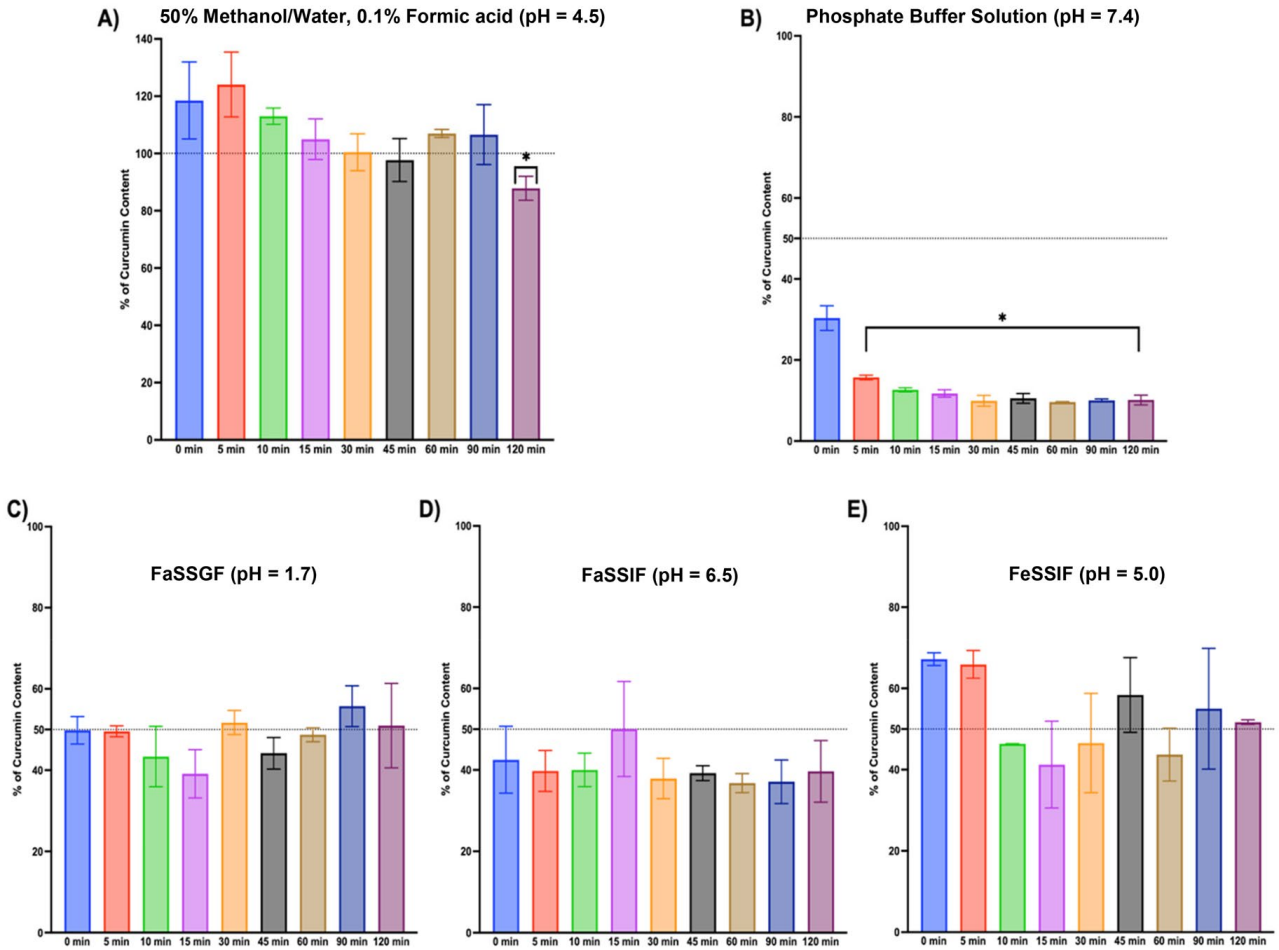
Curcumin stability in (A) 50:50 methanol/water (0.1% formic acid), (B) phosphate buffer solution, (C) FaSSGF, (D) FaSSIF, (E) FeSSIF. 100 µM of pure curcumin was incubated in various biorelevant media over 120 min. Each bar represents the remaining amount of curcumin from duplicate samples. (*) indicates statistical significance from a one-way ANOVA with a Dunnett’s multiple comparisons test to compare each incubation time point to the initial (0-time point). Alpha was set to 0.05, and *p* values were adjusted for statistical significance with the number of comparisons (8 total comparisons).

**Figure 2.**
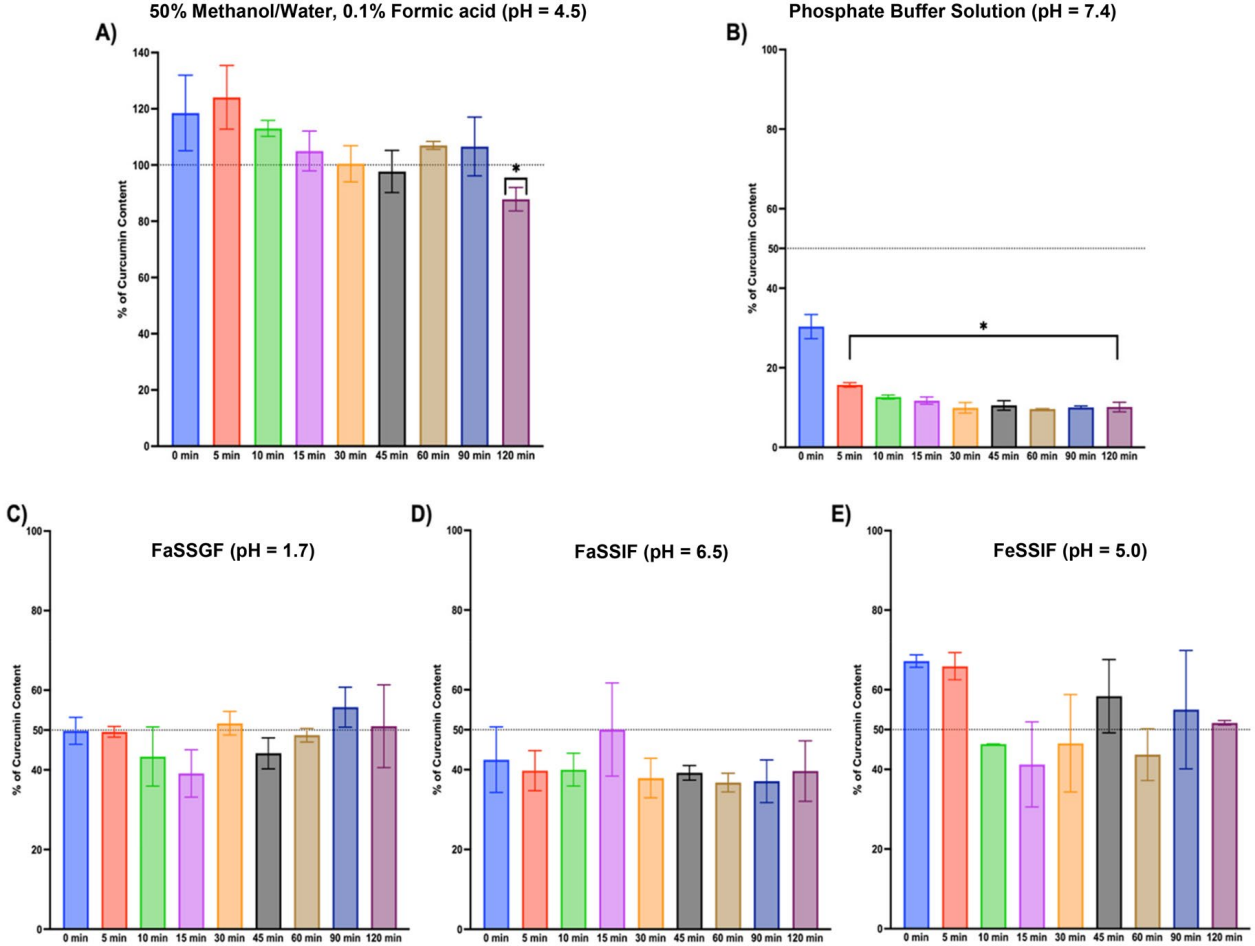
Curcumin stability within ethanolic turmeric extract in (A) 50:50 methanol/water (0.1% formic acid), (B) phosphate buffer solution, (C) FaSSGF, (D) FaSSIF, (E) FeSSIF. 50 µM of curcumin as an ethanolic turmeric extract was incubated in various biorelevant media over 120 min. Each bar represents the remaining amount of curcumin from duplicate samples. (*) indicates statistical significance from a one-way ANOVA with a Dunnett’s multiple comparisons test to compare each incubation time point to the initial (0-time point). Alpha was set to 0.05, and p values were adjusted for statistical significance with the number of comparisons (8 total comparisons).

When incubated in the three biorelevant media – FaSSGF (pH 1.7), FaSSIF (pH 6.5), and FeSSIF (pH 5.0) – both pure curcumin and turmeric extract–derived curcuminoids underwent rapid degradation within the first 10 min. Thereafter, residual concentrations remained relatively stable over the 120-minute incubation period. This biphasic pattern was consistently observed for both pure curcumin (Figure 1) and curcuminoids from turmeric extract (Figure 2), suggesting an initial burst of degradation – likely due to aqueous hydrolysis or oxidative decomposition – followed by a plateau in concentration reflecting solubility limits and low aqueous dispersion.

For pure curcumin, the most favorable retention was seen in FeSSIF and FaSSGF, with approximately 50% of the compound remaining at 2 h. In contrast, FaSSIF (pH 6.5) produced more pronounced degradation, with only ∼40% of the original concentration remaining after 120 min. These findings align with prior studies demonstrating that curcumin undergoes enhanced base-catalyzed hydrolysis at near-neutral pH, owing in part to its enol-keto tautomerism and structural susceptibility to degradation under mildly basic conditions (Wang et al. 1997; Kurita and Makino 2013; Nelson et al. 2017a).

Turmeric extract followed a similar trend, though degradation appeared slightly attenuated, potentially due to matrix effects or protective interactions from co-extracted components. FeSSIF again produced the highest retention (>50%), followed by FaSSGF (∼48%) and FaSSIF (∼45%). While minor, these differences suggest that even under simulated fed-state or fasted-state gastric conditions, curcuminoid degradation remains substantial.

Importantly, comparison to two control matrices – (1) a 50:50 methanol:water solution acidified with 0.1% formic acid, and (2) phosphate buffer at pH 7.4 – confirmed the instability profile of curcumin in aqueous systems. The methanol:water mixture preserved curcumin nearly completely (>95% remaining at 120 min), consistent with curcumin’s chemical stability in organic/acidic environments. In contrast, phosphate buffer at pH 7.4 yielded the most rapid and extensive degradation (>90% lost within 60 min), mirroring the observations reported by others and reaffirming curcumin’s vulnerability to hydrolysis at physiological pH in fully aqueous systems (Wang et al. 1997).

Collectively, these results indicate that while biorelevant media better approximate *in vivo* gastrointestinal conditions than simple buffers, they do not prevent degradation of curcuminoids – especially under near-neutral intestinal conditions (e.g., FaSSIF). The acidified methanol control highlights the compound’s inherent chemical stability when protected from aqueous hydrolysis, whereas the phosphate buffer underscores the impact of pH and solvent environment on curcumin stability.

### Disintegration in biorelevant media

Disintegration testing was conducted on eight commercially available turmeric dietary supplement products using four biorelevant media (FaSSGF, FaSSIF, FeSSIF, FeSSGF). Results are summarized in Table 5. According to USP General Chapter ⟨2040⟩, dietary supplement dosage forms are expected to disintegrate fully within 30 min unless otherwise specified by the monograph (USP <2040>). Using this criterion, six of the eight products passed in all four media, with five products achieving complete disintegration within 15 min.

**Table 5.** Disintegration times (min.) of eight commercially available turmeric dietary supplements in three biorelevant media (900 mL, 37 °C).

Product	FaSSGF (pH 1.7)	FaSSIF (pH 6.5)	FeSSIF (pH 5.0)	FeSSGF (pH 4.5)
4295PR	10 min.	5 min.	2 min.	<15 min.
4296PR	6 min.	6 min.	9 min.	<15 min.
4297PR	10 min.	15 min.	12 min.	<30 min.
4299PR	20 min.	20 min.	20 min.	<30 min.
4663PR	15 min.	15 min.	15 min.	<15 min.
4664PR	40 min.	26 min.	40 min.	<30 min.
4818PR	7 min.	7 min.	8 min.	<15 min.
4819PR	>90 min.	>90 min.	>90 min.	>90 min.

Disintegration times represent the time to complete disintegration for each product; values >30 min indicate incomplete disintegration during the testing window. Due to the opacity of FeSSGF, disintegration times were evaluated in 15 min. intervals.

4664PR exhibited prolonged disintegration times of 40 min in both FaSSGF and FeSSIF, thus failing in those two media, though it passed in FaSSIF and FeSSGF. These extended times may reflect either a delayed hydration phase or stabilizing interactions between formulation excipients and the acidic/colloidal components of the fed-state media. Notably, the capsule did ultimately disintegrate without visible residue in those cases.

4819PR failed to fully disintegrate in any of the four biorelevant media, even after 90 min of testing. In all replicates, remnants of undissolved turmeric extract and portions of the capsule shell remained within the baskets at the final timepoint. The lack of complete disintegration in both gastric and intestinal models suggests that this formulation may be resistant to breakdown under physiologically simulated conditions. Possible contributing factors include a cross-linked or polymer-embedded matrix, hydrophobic coatings, or other delivery technologies that may delay release.

All testing was performed at 37 °C using fluted disks to ensure complete immersion and to prevent capsule flotation. FeSSGF’s visual opacity necessitated inspection of the baskets at 15-minute intervals. Taken together, these findings emphasize the variability in disintegration performance across turmeric formulations and highlight the importance of evaluating dosage form behavior under both fasted and fed conditions that mimic the gastrointestinal environment.

### Dissolution and bioaccessibility performance

**Figure 3.**
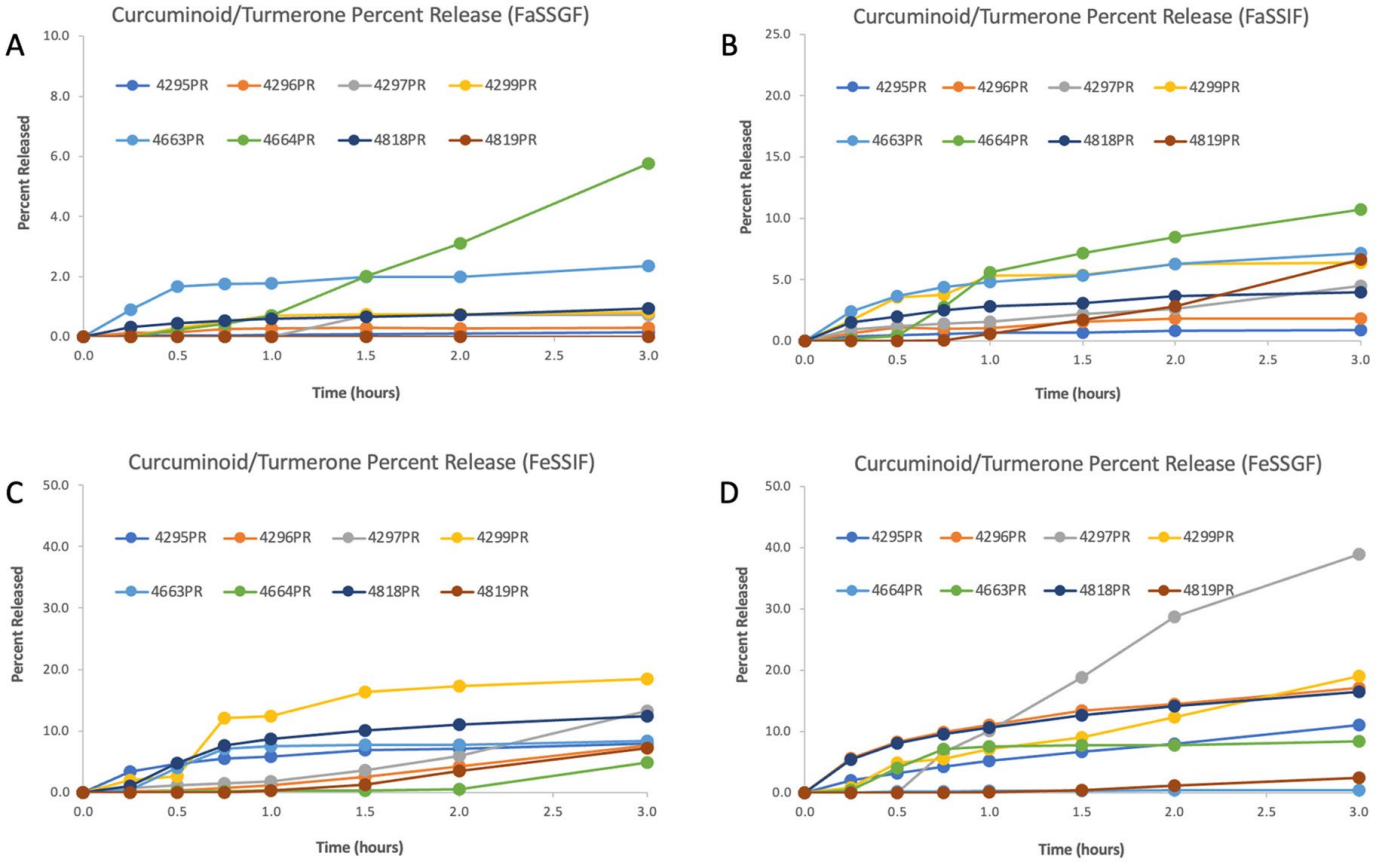
Curcuminoid/(S)-ar-turmerone percent release profiles for eight curcumin-containing dietary supplements in biorelevant media: FaSSGF (A), FaSSIF (B), FeSSIF (C), and FeSSGF (D). Y-axes are scaled differently to highlight product differences.

**Figure 4.**
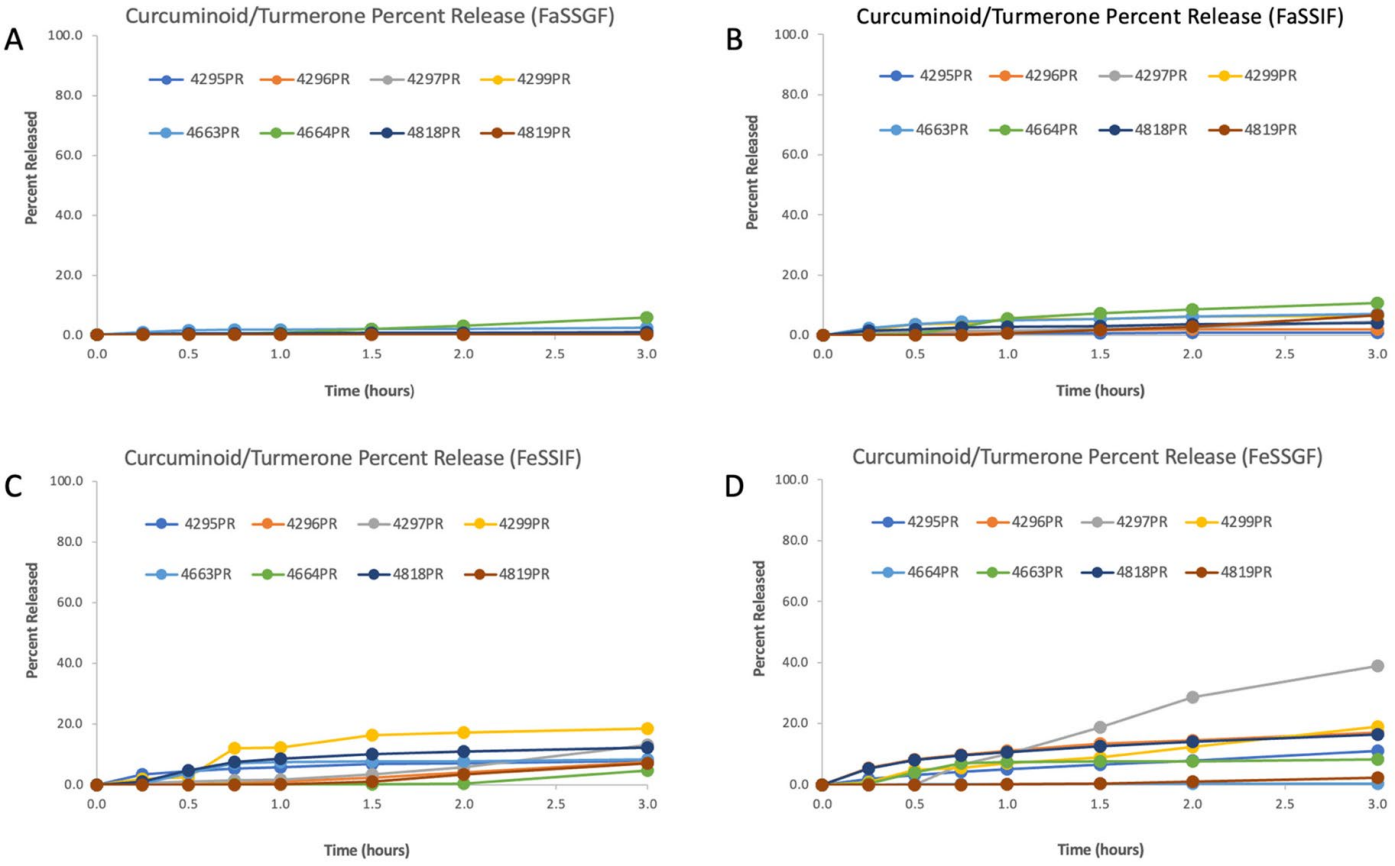
Curcuminoid/(S)-ar-turmerone percent release profiles for eight curcumin-containing dietary supplements in biorelevant media: FaSSGF (A), FaSSIF (B), FeSSIF (C), and FeSSGF (D). Y-axes are scaled to 100%.

The dissolution performance of all eight commercial turmeric products was uniformly poor across the four biorelevant media (FaSSGF, FaSSIF, FeSSIF, and FeSSGF, with no product achieving greater than 40% total release of curcuminoids or (S)-ar-turmerone after 3 h of testing. As shown in Figures 3 and 4, dissolution was consistently lowest under fasted-state conditions, particularly in FaSSGF and FaSSIF, where most products released less than 10% of their total content. Several products – including 4819PR and 4664PR – released less than 2% under these conditions, despite being marketed as bioavailability-enhanced formulations.

Fed-state conditions modestly improved dissolution performance, with FeSSGF representing the most permissive matrix. Under these conditions, the 4297PR phytosome formulation demonstrated the highest observed release of any product, achieving 38.97% total release after 3 h (Figures 3 and 4; Table 9). 4296PR and 4299PR also exceeded 15% release in FeSSGF, whereas 4819PR achieved only 2.40%, and 4463PR and 4464PR failed to surpass 9% release in any medium. Across products, FeSSGF consistently outperformed FeSSIF, consistent with the greater lipid content and emulsification capacity of fed-state gastric fluid relative to fed-state intestinal fluid, as opposed to the modest difference in pH between the two media.

Tables 6–8 further contextualize these findings by reporting bioaccessible concentrations and total quantities of curcuminoids and (S)-ar-turmerone after 3 h of dissolution. In contrast to rankings based solely on percent release or dose fraction, the highest bioaccessible curcuminoid concentrations in FeSSIF and FeSSGF were observed for 4818PRand 4295PR, followed by 4663PR and 4297PR. This pattern reflects differences in total curcuminoid content per capsule (Table 3), as 4818PR, 4296PR, and 4663PR contained substantially higher curcuminoid loads than 4297PR. Accordingly, products with relatively poor release efficiency were still capable of generating higher absolute bioaccessible concentrations when the initial curcuminoid dose was large. In contrast, 4297PR achieved comparatively high bioaccessible concentrations despite having the lowest curcuminoid content per capsule, consistent with its superior release profile (Figures 3 and 4; Table 9).

**Table 6.** Bioaccessible concentrations of curcuminoids (µg/mL) after 3 h in biorelevant media (mean ± SD).

	4295PR	4296PR	4297PR	4299PR	4663PR	4664PR	4818PR	4819PR
**FaSSGF**	0.83 ± 0.26	0.33 ± 0.06	0.7 ± 0.01	0.97 ± 0.03	10.45 ± 1.93	6.71 ± 0.95	2.48 ± 0.98	ND
**FaSSIF**	4.48 ± 0.14	2.29 ± 1.71	4.36 ± 1.97	6.27 ± 0.30	31.73 ± 5.00	12.46 ± 0.89	12.29 ± 2.18	9.78 ± 6.63
**FeSSIF**	43.47 ± 5.35	10.10 ± 1.38	12.86 ± 1.12	25.72 ± 3.04	37.23 ± 4.28	5.58 ± 0.13	50.73 ± 3.33	10.59 ± 5.02
**FeSSGF**	55.02 ± 0.59	23.05 ± 1.28	37.41 ± 6.56	20.44 ± 7.64	37.45 ± 2.90	0.44 ± 0.05	59.41 ± 17.43	3.55 ± 1.16

ND = not detected.

**Table 7.** Bioaccessible concentrations of (S)-ar-turmerone (µg/mL) after 3 h in biorelevant media (mean ± SD).

	4295PR	4296PR	4297PR	4299PR	4663PR	4664PR	4818PR	4819PR
**FaSSGF**	ND	0.27 ± 0.01	ND	0.36 ± 0.06	ND	ND	2.34 ± 0.34	ND
**FaSSIF**	ND	0.84 ± 0.03	ND	1.97 ± 0.70	ND	ND	8.22 ± 0.93	ND
**FeSSIF**	ND	1.64 ± 0.09	ND	4.32 ± 0.64	ND	ND	13.04 ± 1.16	ND
**FeSSGF**	0.45 ± 0.06	2.13 ± 0.06	0.43 ± 0.03	12.75 ± 2.38	ND	ND	14.76 ± 4.25	ND

ND = not detected.

**Table 8.** Bioaccessible quantity (mg) of curcuminoids + (S)-ar-turmerone after 3 h in biorelevant media (mean ± SD).

	4295PR	4296PR	4297PR	4299PR	4663PR	4664PR	4818PR	4819PR
**FaSSGF**	0.75 ± 0.23	1.14 ± 0.15	0.63 ± 0.01	1.19 ± 0.04	9.33 ± 1.72	5.99 ± 0.85	4.31 ± 0.93	ND
**FaSSIF**	4.03 ± 0.12	6.93 ± 4.64	3.91 ± 1.77	9.20 ± 3.49	28.33 ± 4.46	11.13 ± 0.79	18.30 ± 2.01	8.74 ± 5.92
**FeSSIF**	39.12 ± 4.81	28.75 ± 3.67	11.58 ± 1.00	27.04 ± 2.43	33.24 ± 3.82	4.98 ± 0.12	56.94 ± 3.79	9.46 ± 4.48
**FeSSGF**	50.38 ± 1.11	64.15 ± 3.15	34.06 ± 5.93	26.47 ± 6.64	33.44 ± 2.59	0.38 ± 0.04	75.61 ± 2.19	3.17 ± 1.04

ND = not detected.

**Table 9.** Bioaccessible dose fraction (% release) of curcuminoids + (S)-ar-turmerone in biorelevant media after 3 h.

	4295PR	4296PR	4297PR	4299PR	4663PR	4664PR	4818PR	4819PR
**FaSSGF**	0.15%	0.30%	0.72%	0.81%	2.35%	5.77%	0.94%	---
**FaSSIF**	0.82%	1.84%	4.47%	6.28%	7.15%	10.73%	3.97%	6.62%
**FeSSIF**	7.97%	7.62%	13.25%	18.46%	8.39%	4.80%	12.36%	7.16%
**FeSSGF**	10.26%	16.99%	38.97%	18.07%	8.44%	0.37%	16.42%	2.40%

Dose fraction = (bioaccessible curcuminoid + AR-turmerone quantity)/(total curcuminoid + AR-turmerone content per capsule) x 100.

Normalization of these data as bioaccessible dose fractions (Table 9) did not alter the overall ranking of product performance but highlighted the magnitude of the shortfall relative to label claims. Only the 4297PR phytosome formulation exceeded 30% dose fraction in any matrix, while most products ranged between approximately 0.3% and 18% depending on the medium. Fasted-state matrices were least effective overall, whereas FeSSGF consistently yielded the highest dose fractions across products.

Collectively, these results demonstrate that curcuminoid bioaccessibility from commercial turmeric supplements is constrained by a combination of limited dissolution efficiency and formulation-dependent dose loading. Products with higher curcuminoid content per capsule can generate greater bioaccessible concentrations despite poor release profiles, whereas formulations with superior release efficiency may still deliver lower absolute amounts when the initial curcuminoid load is small.

## Discussion

The results of the present study provide the first systematic evaluation of both disintegration and biorelevant dissolution behavior across a cross section of commercially available turmeric dietary supplements. Although numerous prior investigations have examined curcumin dissolution using individual formulations or experimental delivery systems, no previous study has directly compared multiple marketed turmeric products under standardized fasted- and fed-state biorelevant media. By jointly assessing dosage-form disintegration and curcuminoid dissolution, the present work addresses a critical gap between formulation design claims and fundamental *in vitro* performance.

When viewed in the context of earlier dissolution studies, the findings reported here are broadly consistent with prior observations that curcumin exhibits limited and highly variable release in aqueous and biorelevant media. Studies using USP dissolution apparatus have demonstrated poor dissolution of crystalline curcumin, with strong dependence on pH and surfactant presence (Rahman et al. 2009). Evaluations of nanoparticulate curcumin systems have shown only formulation-dependent and often incomplete improvements in dissolution behavior (Ravichandran 2013). Investigations of commercially available turmeric capsules have similarly reported low and inconsistent curcuminoid release under simulated gastrointestinal conditions (Monton et al. 2016). More recent work examining eutectic mixtures and alternative solvent systems has demonstrated enhanced solubility in select systems, though dissolution remains highly media-dependent (Jeliński et al. 2019). Solid dispersion approaches have likewise produced variable dissolution outcomes that depend strongly on carrier composition and drug loading (Setyaningsih et al. 2021), while lipid-based dispersion systems evaluated in biorelevant media have shown improved but still incomplete curcumin release profiles (Song et al. 2022). The present study extends these findings by demonstrating that such limitations persist across commercially available products, including those marketed as bioavailability-enhanced formulations.

The results of this study highlight several persistent challenges associated with curcumin oral bioavailability, despite the diversity of commercially available products employing a range of formulation strategies. Across all eight products tested, none achieved more than ∼40% curcuminoid release in any biorelevant medium after three hours, with the majority falling well below this threshold. Even among those products marketed as novel formulations with purportedly enhanced absorption profiles, *in vitro* dissolution remained suboptimal. These findings are consistent with prior dissolution studies demonstrating poor aqueous solubility and limited curcumin release in both conventional buffer systems and biorelevant media (Rahman et al. 2009; Monton et al. 2016; Song et al. 2022). Importantly, the present results further demonstrate that dissolution performance is markedly improved under fed-state gastric conditions, underscoring the influence of dietary fat and emulsified lipid phases on curcuminoid solubilization.

Curcumin’s inherently low aqueous solubility (approx. 11 ng/mL in water), together with its rapid chemical degradation under physiological conditions – particularly at neutral or mildly basic pH – severely limits its bioaccessibility. Early stability studies demonstrated that curcumin undergoes rapid decomposition in phosphate buffer near physiological pH, while exhibiting substantially greater stability under acidic conditions or in protein-containing matrices (Wang et al. 1997). In the present study, curcumin degradation was particularly pronounced in FaSSIF (pH 6.5), consistent with this well-established pH dependence. This behavior was observed whether curcumin was administered as a pure compound or as part of a turmeric extract. By contrast, the modest protection observed in acidic and fed-state media – including FaSSGF (pH 1.7), FeSSGF (pH 4.5), and FeSSIF (pH 5.0) – reflects both pH-dependent stability and the enhanced solubilization afforded by lipid-containing fed-state gastric media. (Song et al. 2022).

A practical implication of these findings is that turmeric-containing dietary supplements are likely to achieve greater curcuminoid bioaccessibility when administered with food, preferably a fat-containing meal. In the present study, fed-state gastric conditions (FeSSGF) consistently produced higher dissolution, bioaccessible concentrations, and dose fractions than fasted-state or fed-state intestinal media for all products tested. Despite this, only three of the eight products evaluated (4295PR, 4296PR, and 4819PR) include label recommendations to take the product with food. The absence of such guidance on most product labels may therefore contribute to attenuated real-world performance relative to the dissolution behavior observed under fed-state conditions *in vitro*.

Compounding these chemical limitations are formulation-dependent issues related to basic dosage-form performance. Disintegration testing revealed that two of the eight products evaluated (4664PR and 4819PR) failed to disintegrate within the 30-minute interval specified for immediate-release botanical dietary supplements under USP ⟨2040⟩, with one product remaining intact even after 90 min, particularly in FaSSIF and FeSSIF media. Inadequate or delayed disintegration inherently restricts the effective surface area available for dissolution, providing a mechanistic explanation for the low curcuminoid release observed for these products despite marketing claims of enhanced delivery. These findings underscore the importance of evaluating fundamental physicochemical performance attributes – such as disintegration behavior and chemical stability – before the potential benefits of more complex formulation technologies can be meaningfully interpreted, consistent with the performance expectations outlined in the USP–NF *Curcuminoids Capsules* and *Curcuminoids Tablets* monographs (USP-NF, Curcuminoids Capsules; USP-NF Curcuminoids Tablets).

The 4819PR formulation is especially illustrative of this disconnect between *in vitro* performance and reported *in vivo* exposure. In a simulated gastrointestinal model, 4819PR exhibited comparatively high curcumin bioaccessibility (i.e., curcumin in the mixed micelle phase) relative to other commercially available supplements (Zheng et al. 2018). *In vivo*, 4819PR has also been used as a comparator in preclinical pharmacokinetic work evaluating engineered lipid-based delivery systems, underscoring its role as a benchmark “enhanced” commercial formulation (Gupta et al. 2020). In human subjects, a related commercial iteration (4819PR Ultra) produced substantially higher systemic exposure to total curcuminoids – which largely reflect circulating conjugated metabolites – than a conventional 95% turmeric extract under fasting conditions (Kothaplly et al. 2022). In the present study, the 4819PR lot evaluated exhibited poor *in vitro* disintegration and dissolution under both fasted- and fed-state conditions, a finding that would be expected to limit curcuminoid bioaccessibility and, consequently, systemic exposure even when circulating conjugated metabolites are the primary analytes. While such behavior does not necessarily preclude absorption *in vivo* – particularly in the presence of bile salts, surfactant-containing intestinal fluids, or extended gastrointestinal residence – these data challenge the assumption that superior release properties observed *in vitro* are invariably accompanied by enhanced systemic exposure. Moreover, there remains ongoing debate regarding whether conjugated curcuminoids are themselves biologically active. While some evidence suggests that deconjugation at tissue sites could permit localized regeneration of free curcumin, thereby enabling site-specific activity (Kunihiro et al. 2019), substantial uncertainty remains regarding the pharmacodynamic relevance of curcumin glucuronides and sulfates in systemic circulation (Nelson et al. 2017b). Critical evaluations of curcumin pharmacology have emphasized that increased plasma concentrations of conjugated metabolites do not necessarily translate into meaningful biological activity at distant tissue sites (Nelson et al. 2017a). This uncertainty calls into question the clinical significance of so-called ‘bioavailability enhancements’ that primarily reflect elevated circulating conjugate levels without demonstrable delivery of free curcumin.

In light of this, it is notable that some turmeric extracts contain other constituents –particularly sesquiterpenes such as (S)-ar-turmerone – that may exert synergistic or absorption-modulating effects. *In vitro* studies using intestinal epithelial models have demonstrated that aromatic and α-turmerone can influence curcumin transport and modulate P-glycoprotein (ABCB1) activity, thereby altering curcumin cellular uptake (Yue et al. 2012). In the present study, turmerone content varied widely across products, with no detectable quantities in 4819PR and over 20 mg per capsule in 4297PR’s phytosome formulation. However, whether such transporter-level effects observed *in vitro* translate into meaningful enhancements of curcumin absorption *in vivo* remains uncertain and has not been systematically established. Nevertheless, sesquiterpenes present in traditional turmeric extracts may offer pharmacokinetic or pharmacological advantages distinct from, or complementary to, synthetic excipient-based delivery strategies (Orellana-Paucar and Machado-Orellana 2022).

The inclusion of piperine in curcumin formulations has historically been motivated by early pharmacokinetic observations suggesting enhanced curcumin exposure following co-administration (Shoba et al. 1998). That study played a pivotal role in shaping subsequent formulation strategies and product labeling, and piperine continues to be incorporated into a number of commercial turmeric supplements. However, more recent controlled human pharmacokinetic studies have yielded mixed results, with several investigations failing to demonstrate a consistent enhancement of unconjugated curcumin absorption attributable to piperine (Fança-Berthon et al. 2021; Kroon et al. 2025).

In the present study, only one product (4296PR) listed piperine as a formulation component, and piperine bioaccessibility or release was not evaluated. Accordingly, no conclusions can be drawn regarding the contribution of piperine to the observed disintegration or dissolution behavior. Rather, these findings underscore that piperine-based strategies operate through mechanisms distinct from solubility enhancement or dosage-form performance and therefore cannot be inferred from *in vitro* dissolution testing alone.

Overall, the present findings reinforce the multifaceted barriers to effective oral delivery of curcumin, including poor aqueous solubility, rapid chemical degradation, inconsistent dosage-form performance, and extensive first-pass metabolism *via* glucuronidation and sulfation. Although a wide range of formulation strategies – such as micellar systems, nanoparticles, phospholipid complexes, and essential oil–based preparations – have been developed to address these limitations, critical evaluations of curcumin pharmacology emphasize that increased systemic exposure does not necessarily reflect improved delivery of biologically relevant free curcumin (Nelson et al. 2017a; 2017b). Consistent with this perspective, the data reported here demonstrate that formulation innovation among commercially available products does not reliably translate into improved *in vitro* disintegration or dissolution performance, particularly under fasted-state conditions that may not reflect typical consumer use.

## Conclusions

Despite its well-documented safety profile and broad range of biological activities observed both *in vitro* and *in vivo*, curcumin remains a challenging compound to deliver systemically following oral administration. Poor aqueous solubility, chemical instability under physiologically relevant conditions, inconsistent dosage-form performance, and extensive first-pass metabolism resulting in predominantly conjugated circulating species collectively limit the availability of free curcumin *in vivo* (Nelson et al. 2017a, 2017b). These well-recognized limitations have motivated the development of numerous formulation strategies intended to enhance curcumin exposure; however, as demonstrated in the present study, improvements in formulation design do not necessarily translate into consistent gains in disintegration or biorelevant dissolution performance across commercially available products, particularly under fasted-state conditions.

An important practical implication of these findings is that turmeric-containing dietary supplements are more likely to achieve meaningful curcuminoid bioaccessibility when administered with food. In the present study, fed-state gastric conditions consistently produced higher dissolution, bioaccessible concentrations, and dose fractions than fasted-state or fed-state intestinal media, reflecting the influence of dietary lipid content on solubilization. Notably, only a minority of the products evaluated included label recommendations to take the supplement with food, raising the possibility that consumption with water alone may limit curcumin exposure relative to fed-state conditions.

This observation raises an important question regarding the therapeutic relevance of conjugated curcumin metabolites. While circulating glucuronide and sulfate conjugates generally exhibit weak activity in conventional *in vitro* assays, accumulating evidence indicates that their biological effects may arise from localized regeneration of the parent aglycone at sites of inflammation through the action of β-glucuronidase enzymes released by activated immune cells (Perez-Vizcaino et al. 2012; Kawai 2018). This conjugation–deconjugation paradigm, often described as the ‘flavonoid paradox,’ has been extensively characterized for flavonoids such as quercetin and is supported by studies demonstrating vascular, macrophage-mediated, and neutrophil-associated hydrolysis of glucuronidated metabolites in inflamed tissues (Bartholomé et al. 2010; Perez-Vizcaino et al. 2012; Ishisaka et al. 2013; Sheridan and Spelman 2022; Kroon et al. 2025).

Although curcuminoids are structurally distinct from classical flavonoids, curcumin-β-D-glucuronide and related metabolites have likewise been shown to undergo β-glucuronidase-mediated hydrolysis *in vivo*, resulting in localized liberation of free curcumin within tissues (Kunihiro et al. 2019, 2020). These findings suggest that differences in conjugate formation, absolute dose delivered, and tissue-specific deconjugation may influence the pharmacological relevance of curcumin formulations, even when systemic concentrations of free curcumin remain low.

From a mechanistic perspective, the presence of non-curcuminoid constituents such as (S)-ar-turmerone in whole turmeric extracts highlights an additional layer of complexity in evaluating curcumin delivery strategies. Such constituents may contribute secondary pharmacokinetic or biological effects that are not captured by dissolution testing of isolated curcuminoids. While experimental studies and critical reviews have suggested potential roles for turmeric oil sesquiterpenes in modulating intestinal transport, their quantitative contribution to curcumin absorption and efficacy in humans remains uncertain (Yue et al. 2012; Orellana-Paucar and Machado-Orellana 2022). These considerations highlight the limitations of conventional bioavailability assessments and the importance of evaluating turmeric formulations as chemically complex systems rather than simple single-compound products.

Taken together, these findings underscore the need for careful interpretation of curcuminoid “bioavailability” claims. Increases in systemic concentrations of conjugated metabolites do not necessarily translate into therapeutic efficacy unless those conjugates are biologically active or undergo reliable, tissue-specific deconjugation at sites of action. Future work should prioritize validating the extent of *in situ* regeneration of curcumin aglycone under inflammatory conditions in humans, characterizing tissue-level β-glucuronidase activity, and clarifying the pharmacodynamic relevance of conjugated versus unconjugated curcuminoids *in vivo*.

## Data Availability

The data that support the findings of this study are available from the corresponding author, BJG, upon reasonable request.
